# In memoriam of Huaxi Xu, PhD, 1964–2022

**DOI:** 10.1186/s13024-022-00587-z

**Published:** 2023-01-26

**Authors:** Guojun Bu, Hui Zheng

**Affiliations:** 1 Molecular Neurodegeneration, Jacksonville, FL 32224 USA; 2 Huffington Center On Aging, Baylor College of Medicine, Houston, TX 77030 USA

In the spring of 2005, when ***Huaxi*** (Xu) and (Guojun) ***Bu*** met up during a meeting, their discussion on scientific publishing in the neurodegenerative disease field led to a realization that there had not been a journal that was devoted to addressing the molecular and cellular aspects of neurodegenerative disease mechanisms. Thus, the idea of creating a new journal was conceived. In 2006, *Molecular Neurodegeneration*, an open access journal published by BioMed Central, started publishing with Bu and Huaxi as Co-Editors-in-Chief. Seventeen years later, *Molecular Neurodegeneration* has become a leading open-access neuroscience journal.

On October 14, 2022, Dr. Huaxi Xu passed away after his brave battle against cancer. He was simply too young to leave us, but the legacy he left behind is so robust that we will remember and honor him forever.
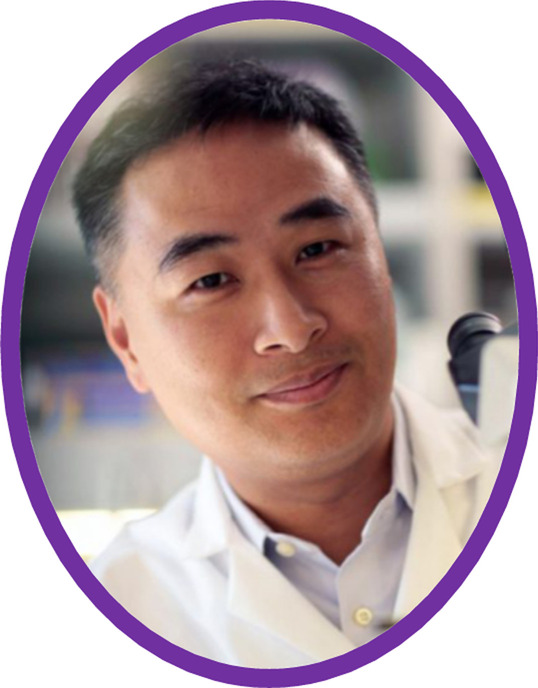


Dr. Xu’s early training during his PhD included areas of biochemistry, cell biology and neuroscience at Albert Einstein College of Medicine (1988–1993) where he was co-mentored by Dennis Shields and Nobel laureate, Gunter Blobel, investigating the mechanisms underlying proteolysis and trafficking of membrane and secretory proteins. Dr. Xu then pursued postdoctoral training with another Nobel laureate, Paul Greengard at Rockefeller University (1994–1998), where he joined the Alzheimer’s disease (AD) program co-directed by Greengard and Sam Gandy, now a Professor at the Icahn School of Medicine at Mount Sinai. Together with Greengard and Gandy, Huaxi defined subcellular mechanisms underlying APP processing and consequent generation of the pathogenic amyloid-β (Aβ) peptide in AD. Importantly, his postdoctoral research led to seminal findings describing the regulatory mechanisms for Aβ generation occurring via signal transduction pathways involving PKA, PKC, Abl kinase and insulin that controlled sorting of APP through intracellular compartments such as the endoplasmic reticulum, trans-Golgi network, and endosomal-lysosomal system. One milestone finding was that estrogen can reduce Aβ generation, providing a potential mechanistic link between menopause and a higher risk for developing AD.

Dr. Xu’s research as an independent investigator at the Sanford-Burnham Prebys Medical Discovery Institute (2003–2020) expanded further, leading him to explore new mechanisms related to tau pathogenesis, novel molecular links between AD and Down’s syndrome (DS), and groundbreaking findings related to tauopathy, and neuroinflammation/glial biology. Dr. Xu’s most recent work focused on addressing how a microglia-specific gene, *TREM2*, impacts AD risk. His group was the first to identify Aβ as an endogenous ligand for TREM2, providing mechanistic insight as to how *TREM2*-expressing microglia are attracted to the Aβ plaque. Their multi-omics comparison of AD variants including those of *TREM2* in human embryonic stem cell-derived microglia revealed a pathway converging on *APOE*, the strongest genetic risk factor for AD. Together, the scope of Dr. Xu’s work has been extensive, but his observations have consistently funneled into a focused understanding of how various pathways are interconnected in multiple neurodegenerative disorders. His most recent appointments include a professorship at Xiamen University as well as a distinguished professorship and directorship of the Institute for Brain Science and Disease at Chongqing Medical University.

Dr. Huaxi Xu was an undisputed leader in the field of AD research. Throughout his prolific career, he published more than 200 research and review articles. A few representative papers are listed at the end of the article [1–15]. His work was consistently of high-quality and generated sustained impact in the field of neurodegenerative disease research in general, especially in AD. Dr. Xu had a strong reputation of being collegial in scientific collaboration and generous as a mentor. He was passionate about bringing scientists together to debate the leading pathogenic mechanisms and address complex questions as a team.

We invited his son Raymond Xu and a few of his trainees and colleagues to share their own perspectives in memory of Dr. Huaxi Xu.

**Raymond Xu (** ***Wake Forest Baptist Medical Center*** **)**

I am so proud of my dad. He fought courageously in his battle with cancer until the very end, which for anyone who knew him was not a surprise.

He immigrated to America over 30 years ago to pursue a higher education and to embrace the country’s academic opportunity. My fondest and earliest memories of my father were of weekends spent in the laboratory at Rockefeller University, where he would show me colorful histology slides and how to handle mice. Soon after the experiments were finished, we would rush over to Madison Square Garden to catch the evening Knicks game. On the subway ride, I would ask why he worked so hard so as to spend his Saturdays in a lab coat. “Because son, knowledge is power,” pointing to his Francis Bacon t-shirt. While I did not understand the gravity behind those words at seven years old, seeing his dedication to science made a lasting impression.

Being alongside my father in his final weeks, I saw how loved he was by his family and friends. It was clear he was not only a great scientist, but also a great friend. To me, he was great father. He showed me the meaning of hard work, while still making time to enjoy a basketball game.

**Dongming Cai (** ***Icahn School of Medicine at Mount Sinai*** **), Wenjie Luo (** ***Weill Cornell Medicine*** **), Hong Wang (** ***Eli Lilly and Company*** **) and Ping Han (** ***Merck and Company*** **)**

Like many others, Huaxi’s passing brought us unbearable sadness. Huaxi was an inspirational scientist, a great mentor and colleague, and a big brother to us.

We met Huaxi during our postdoctoral training in Dr. Paul Greengard’s lab at Rockefeller University in the early 2000s. As the AD group leader, Huaxi was passionate about understanding cellular mechanisms underlying AD pathogenesis. Applying his cell biology expertise, Huaxi led the effort to carefully examine and dissect the biosynthesis and intracellular trafficking of several AD molecules such as APP, PS1 complex and BACE1. He was a pioneer who developed and optimized many novel approaches applied in the AD field including vesicle budding assays and cell surface protein labeling techniques.

As the first group of postdoctoral fellows trained by Huaxi, we had the opportunity to work with him closely. He guided us into the AD research, taught us top notch benchwork techniques, and worked with us side-by-side on many interesting projects. His passion and enthusiasm for science greatly inspired us. We can vividly recall moments when Huaxi couldn’t stop talking about a new idea he had with sparks in his eyes. Huaxi was a great mentor and colleague, connecting us with many superb scientists around the world through his social network. He always encouraged us to advance in our careers and remained supportive and approachable whenever we needed. He was also a big brother who cared with patience and love, often sharing his experience of balancing life as a scientist and as a parent.

We are among many whose careers were nurtured by Huaxi. His scientific spirit, shining character, and unconditional support to friends and colleagues touched so many hearts. Huaxi was irreplaceable and will be remembered forever.

**Yun-Wu Zhang (** ***Xiamen University*** **)**

I was one of the earliest postdoctoral fellows recruited by Huaxi at the Sanford-Burnham-Prebys Medical Discovery Institute in 2003, when he had just moved from Rockefeller University. A few years later, under Huaxi’s guidance we established the Institute of Neuroscience at Xiamen University where I currently serve as the director. As a long-term student, friend, and colleague of Huaxi, I had the honor to witness Huaxi grow from a vigorous young talent to an internationally recognized expert in AD research through his perseverance and hard work. I am saddened that, at the peak of his career, Huaxi left us after a brief battle with cancer.

In his short but extraordinary life, Huaxi has published hundreds of high-quality papers. These achievements have greatly promoted our understanding of the molecular mechanism underlying AD and laid a solid foundation for drug development.

As well, Huaxi was an excellent teacher. He mentored many outstanding students from all over the world. Now, many of them are following his footsteps. His meticulous, diligent, and conscientious attitude towards scientific research will leave a long-lasting influence on his trainees. Huaxi was a joyful person who loved making friends. He treated people sincerely and warmly and was always willing to help with all his heart.

The happy times we spent with Huaxi cannot come again. But we shall never forget him.

**Timothy Huang (** ***Sanford Burnham Prebys Medical Discovery Institute*** **)**

Huaxi was my postdoctoral mentor at Sanford Burnham (now SBP), where I would later establish a research program within his group as a Research Assistant Professor and continue my own program after Huaxi’s return to China prior to the COVID19 pandemic (2020). Huaxi was a most transformative mentor for me, as well as for many others that came through his lab. He embodied a relentless pursuit of perfection, boundless energy and enthusiasm for science, and extreme focus and dedication to his work which he also expected from members of his group. His scientific insight and intuition were always on point, and most importantly he was able to cultivate the potential in his trainees, as evidenced by the successful careers of his many students and postdocs. Huaxi had a friendliness, charm, and charisma that brought everyone together and bound many in rare friendships that would echo throughout a lifetime. In my last text message to Huaxi a few days before he passed away, I said “…most importantly, thank-you for being my friend.” I wish I had added he will always be a “1” (highest NIH ranking score) in “Significance,” “Innovation”, “Investigator”, “Approach” and “Friendship” categories. Knowing Huaxi, he would have likely said I was being too generous.

**Henrietta Nielsen (** ***Stockholm University, Molecular Neurodegeneration*** **)**

Huaxi’s exceptional dedication to science and his nature as a hard-working scientist resulted in an extraordinary research career with numerous important contributions to the field of neurodegeneration. His early passing left a great void in the field of neurodegeneration research and resulted in broken hearts amongst family members and his many friends and colleagues, including mine. Huaxi’s and my paths first crossed ten years ago (2012) when I was recruited by Dr. Guojun Bu as the senior editor of *Molecular Neurodegeneration* for which Bu and Huaxi served as co-editors-in-chief. His dedication to the journal struck me as the selfless dedication of a parent to their child with frequent communications to assess the status, progress, and growth of the journal that over the years has grown to be a leading journal in the field of neuroscience. I deeply value his guidance and leadership along the way, and I will always remember him as an energetic and personable man with an open mind and a big smile. Huaxi, may your family find comfort in knowing how many people’s hearts you’ve touched and thank you for being part of my own journey.

**Hongmei Li (** ***Molecular Neurodegeneration*** **)**

I started having frequent email and phone interactions with Huaxi upon joining *Molecular Neurodegeneration* as a senior editor in 2016, the journal Huaxi and his “brother” in science, Dr. Bu, co-founded in 2006. What impressed me the most about Huaxi was how straightforward he was, encouraging me to speak my mind on various issues we encountered running the journal. I felt his sincerity in helping me learn and improve as an editor, and his passion in building this journal to the highest caliber. I had never imagined he would leave us so soon! His sunny and brave smiles in the recent photos as he was fighting against cancer had kept me hoping he would come back to us. He left us many beautiful and vivid memories, which we will cherish forever. I would like to believe that his spirit is still with us, cheering us on forever.

**Diane Bovenkamp (** ***BrightFocus Foundation*** **)**

BrightFocus is devastated to hear of the passing of Dr. Huaxi Xu. He was a member of our scientific alumni network, past grantee, co-founder of our official journal, *Molecular Neurodegeneration*, and one of the catalysts for the start of the International Society for Molecular Neurodegeneration (ISMND). He was taken from us too soon, but his memory will go on, especially by our collective continuation of support for and participation in MN and ISMND initiatives and conferences.

**Wen‑Biao Gan (** ***ShenZhen Bay Laboratory*** **)**

Huaxi and I met for the first time in New York City in 2000 and we became good friends immediately. I greatly miss our many lunch conversations with him in midtown Manhattan and Chinatown. He was sincere, smart, confident, held high standards for himself and trainees, and practical but with big dreams. Huaxi had this rare combination of traits that engaged and drew people around him. In the past two decades, his character did not change a bit, except that his scientific knowledge augmented tremendously. Huaxi was a veteran and natural leader in the field of AD and was poised to make a real difference after he returned to China. I believed in him, and we had planned to work together closely in the coming years. Unfortunately, his dream was cut short too early and too abruptly. I lost a great friend and brother. Huaxi is simply irreplaceable in life and science, and he will be forever missed.

**Sam Gandy (** ***Icahn School of Medicine at Mount Sinai*** **)**

I first met Huaxi Xu when I was in Paul Greengard’s Lab at Rockefeller working on the cell biology of APP sorting and processing. Huaxi joined Paul’s lab as a postdoctoral fellow after finishing his PhD with the late Dennis Shields at Einstein, and later moved with me to my first independent lab at Cornell. While training with Dennis, Huaxi demonstrated an incredible knack for vesicle biology, and his first contribution to the APP processing story was to reconstitute key *trans*-Golgi network vesicle budding in a cell-free in vitro system. We went on to publish 8 papers together, all focusing on APP and Aβ. Huaxi’s first independent lab was at the Sanford-Burnham Institute in San Diego. From that lab, Huaxi’s output exploded in all directions. Though his focus remained on AD, Huaxi made important contributions to elucidating the function of many molecules over the past 20 years, including sorting nexin 27, vps35, and TREM2. He never met a molecule that stymied him. He just kept plowing ahead.

Of course, Huaxi was much more than a scientist. He was one of the most optimistic people I’ve known, and he was a dedicated father to his son, Raymond, and his daughters, Bernice, Adele, and Madeline. Huaxi and I remained in touch until his hospitalization, and, even then, Raymond regularly posted photos and videos of Huaxi until his last day. He is already missed by family, friends, and colleagues, especially by Guojun Bu, who was Huaxi’s best friend.

**David Holtzman (** ***Washington University School of Medicine*** **)**

I have very fond memories of Huaxi. The one memory that stands out the most is when I had a chance to see him with his family and close friends from both the United States and Xiamen all together at a meeting in China. He had such a twinkle in his eye for those few days. He was so loved by his family and friends.

**Yue‑Ming Li (** ***Memorial Sloan Kettering Cancer Center*** **)**

I first met Huaxi when he was invited to give an expert view on the processing of APP and secretases at Merck Research Laboratories. I was very impressed with his superb work. Undoubtedly, Huaxi was an extraordinary scientist and has made significant contributions to the field of neurodegeneration and AD. As a close friend, we enjoyed our get-togethers at meetings and other gatherings to talk about life and drinks and share crazy antics. Huaxi was an investigator of distinguished achievement and a great human being with an open heart and colorful life. He will be missed greatly, and his legacy will live on.

**Jie Shen (** ***Harvard Medical School*** **)**

I was stunned by Huaxi's message (in the last days), his voice full of lament and yearning for more, more life, more happiness, and more birthday celebrations. The heaviness has stayed with me, making me ponder upon the finality of life.

Huaxi was so full of life and energy, always trying new things and starting new ventures. The journal *Molecular Neurodegeneration* is a perfect example. Huaxi and Bu had the foresight to establish it in the beginning of the digital publication era and nurtured it tirelessly into a publishing powerhouse. As a friend Huaxi is fun and caring. I have countless fond memories, from the dinners we shared in three continents to our last moment together in a glorious spring day when our families rode a carriage together through the Central Park and afterwards our daughters enjoyed a wonderful moment of feeding carrots to the horse.

Rest in peace, my friend. You lived a full life and achieved a great deal. I will do my best to keep your legacy alive by continuing supporting your life's work.

**Sangram Sisodia (** ***University of Chicago, Chicago*** **)**

My “little brother” Huaxi: I miss you and cannot fathom not having you in my world. You were a man of infinite energy, insight, and happiness. Huaxi and I met soon after he joined Sam Gandy and Paul Greengard’s lab where their focus was on defining the cellular compartments involved in constitutive and regulated processing of APP. This began a long-standing collaboration on APP and presenilin metabolism, but more importantly, forged a beautiful relationship and an enduring friendship. Over the years, he found time to celebrate my birthday, whether in Chicago, Clearwater or Tokyo, and celebrate we did! Huaxi was not one to take findings at face value, but rather, took solace in challenging conventional wisdom. He was undeterred and steadfast in his commitment to AD research. Together with Bu as a co-Editor-in-Chief of the journal *Molecular Neurodegeneration*, he served the scientific community with great aplomb. A wonderful father to beautiful children and a true friend to many whose lives he touched—we are all lucky to have known him. In Dhammapada, Chapter 23, The Monk, Gautama Buddha captured the essence of Huaxi’s existence, “Let him associate with friends who are noble, energetic, and pure in life, let him be cordial and refined in conduct. Thus, full of joy, he will make an end of suffering.”

Rest in peace, my little brother. You will remain in my thoughts forever. Till we meet again.

**Rudolph Tanzi (** ***Massachusetts General Hospital*** **)**

My fondest memories of Huaxi are his bigger than life smile and his never-ending profound insights about science whether talking about them at a meeting, a dinner, or the cocktail lounge. Huaxi was as brilliant as he was a loyal friend, always a devoted collaborator, and all around just a great human being. It is no surprise that two of Huaxi’s mentors (Gunter Blobel and Paul Greengard) went on to become Nobel laureates. Huaxi knew good science when he saw it and then brought it to another level when he became involved. To the credit of Huaxi and Bu, they created a new journal (*Molecular Neurodegeneration*) that in relatively short order earned a greater impact factor than some of the highest journals in the neuroscience field. Huaxi could not help being successful in all that he pursued. But what we will remember most about Huaxi is his kind spirit, boundless energy, incredible friendship and, of course, his infectious smile!! Rest in Peace, my friend.

**Gopal Thinakaran (** ***University of South Florida*** **)**

I was fortunate to have known Huaxi for over 25 years, starting when we were both post-doctoral fellows. Through the years, Huaxi was a great friend and colleague, critiquing and offering help with our experiments and, more importantly, providing constant encouragement. I often watched Huaxi in amazement when he was at his best as a great wheeler and dealer, making seamless connections to help everyone. What I’ll miss most about Huaxi is his phone calls, which invariably start with “Gopal, you are no good…” Our conversation will continue when he’d proceed to give profound advice on all matters—big and small—from intricate details of experiments, and project directions, to career advice. I can’t believe our buddy is gone so suddenly.

**Robert Vassar (** ***Northwestern University Feinberg School of Medicine*** **)**

Dr. Huaxi Xu was a loyal friend and colleague to me for many years. I first met Huaxi at a NIH study section where he approached me to introduce himself. His warmth, friendliness and humor won me over immediately. We soon became close friends, so close in fact that we called ourselves “brothers”! I knew I could count on Huaxi through thick and thin – a very rare commodity in the fast and furious world that we live in.

Huaxi’s legacy spans the entire field of molecular and cellular AD research. Early in his career he worked with Nobel Laureate Paul Greengard on APP processing and trafficking. He was a creative thinker and innovator that revealed the cell biology of presenilin and its role in the production of Aβ that is so critical in AD pathogenesis. His interests covered broad aspects of AD research, including recent studies on TREM2 as an Aβ receptor that influences the behavior of microglia, among others.

We should remember Huaxi as a loyal friend, generous colleague, and creative innovator that advanced our understanding of the molecular and cellular mechanisms of AD. He left us tragically all too soon and he will be missed immensely by all those whose lives he has touched. My sincere condolences and sympathies go out to his family at this very difficult time.

**Rong Wang (** ***BGI Genomics*** **)**

Both Huaxi and I started our AD research career in Rockefeller University back in early nineties. When I first met him, he was not only a Postdoctoral Fellow in Professor Paul Greengard’s laboratory, but also a neighbor as we lived on the same floor in the Faculty House. With the help of Drs. Sam Gandy and Sam Sisodia, I developed an immunoprecipitation mass spectrometry (IP-MS) to study Aβ peptides in 1996. During the same time, Huaxi, with his solid training in cell biology and molecular biology, studied signal transduction pathways in the central nervous system and APP biosynthesis pathway. In the latter studies, we applied the IP-MS and revealed the generation of Aβ in the trans-Golgi network in the apparent absence of vesicle formation, which resulted in our first collaborative publication in 1997. Furthermore, I learned a lot of cell biology techniques from him. Since then, we have published seven research papers together. During that time, we not only collaborated scientifically, but also shared some of our spare time together and had many thoughtful discussions about our research and our lives. After knowing him for many years, I learned that he was a wise man with clear and critical thinking; he was a loyal friend with a big heart and supportive hands; he was an outstanding dedicated scientist with love for his research, his trainees, and his friends and colleagues. I will miss him sorely.

**Weiming Xia (** ***Boston University School of Medicine*** **)**

I did not expect that my New Year greetings to Huaxi during the Chinese Spring Festival 2022 would be my last conversation with him after more than a quarter century of friendship (since 1995). Huaxi and I belong to a generation of college graduates who started our life in the US from scratch three decades ago. Huaxi’s son described this in a social media post: “(My father) immigrated to America over 30 years ago with two hundred dollars in his pocket and a dream of a better life”. As our generation started to build our foundation, Huaxi began to help the next. In 1998, Huaxi and I took the train from Amsterdam to Brussels and spent a day with his friends. He learned that they were facing financial difficulties after leaving China to study in this new country. Hauxi then persuaded them to take some money as a gift from him. Like all newcomers, Huaxi pursued his American dream; he also helped countless students like those in Belgium who endured the same struggles he did 30 years prior, all with the goal to build and work for a better life. Huaxi left a world that is no longer the same without him.

**Riqiang Yan (** ***University of Connecticut*** **)**

I first met Huaxi in 1995 when we both were receiving postdoctoral training at the Rockefeller University and living in the Faculty House. Huaxi was energetic, friendly, and dedicative to his research, and he appeared to be quite knowledgeable in his study of AD pathogenesis. When I started to enter my drug discovery research in AD in early 1998, Huaxi became my first frequent contact for discussion, and he had been my free consultant for solving many puzzles that I had encountered while reading some published studies. I was indebted to him for our effective and fruitful discussions, which helped me in the discovery of BACE1. His expertise in protein trafficking led to our collaborative study on BACE1 trafficking. We showed that the transmembrane domain of BACE1 dictates predominant location of BACE1 in the Glogi/TGN compartments, a study published in JBC with Huaxi and Dr. Greengard as collaborators. For the past twenty-seven years, Huaxi had not only been my long-term colleague in AD research but more like a brother.

**X. William Yang (** ***University of California, Los Angeles*** **)**

I met Huaxi Xu over 25 years ago when we both were at Rockefeller University—he was a postdoc and I was an MD/PhD student. To me, he was first and foremost a great friend and a terrific tennis player. He often called me “little brother” and showed me his brotherly love by beating me in tennis games almost every time. After moving to Southern California in the early 2000s to set up our respective labs each focusing on neurodegeneration, our conversations shifted from sports to AD and Huntington’s disease (HD). I always treasured Huaxi’s insightful feedback on our HD work, and his persistence in finding ways for us to work together on AD. I was extremely grateful that my lab’s first NIH grant on AD research was obtained together with Huaxi’s lab, with him and me serving as joint PIs. Our fruitful collaboration led to discoveries related to the roles of TREM2 in amyloid AD pathogenesis. I remember Huaxi for his passion and accomplishments in AD research, his leadership exemplified as the co-founder of the journal *Molecular Neurodegeneration*, his unwavering support to junior scientists, and most importantly, his unforgettable smile both in real life and symbolized as an Emoji in many of his emails.

**Xiongwei Zhu (** ***Case Western Reserve University*** **)**

While I knew that Huaxi was in poor health in the past few months, it is still hard for me to accept that such a joyful person so full of life left his family and friends so early and leaving a large vacuum in our hearts impossible to fill. I first met Huaxi in 2004 when I attended a NIH study section for the first time. We sat side by side and became friends quickly. In the old days, you did not know other reviewers’ opinion until discussing the application at the meeting. It was tough for a newbie to be at odds with other more seasoned reviewers. Huaxi’s encouragement and caring support helped me gain confidence in myself which I still cherish today. My last meeting, which I recall fondly, was when we shared a room during the SfN meeting in Chicago just before the pandemic. Our discussions of life and family felt like it was just yesterday. Huaxi was a great scientist, a caring friend, an attentive big brother, and a source of inspiration. He will be deeply missed.

As a co-founder and Co-Editor-in-Chief of *Molecular Neurodegeneration*, Huaxi’s contribution to the journal’s meteoric rise cannot be underestimated. But Huaxi was so much more: He was a brilliant scientist, an exceptional colleague, a celebrated mentor, a father of four wonderful children, a dear friend to many and, above all, a kind and warm human being. He lived a full life despite it was tragically cut short. This memoriam is just one small reflection on the life he lived, the lives he touched, and the legacy he left behind.
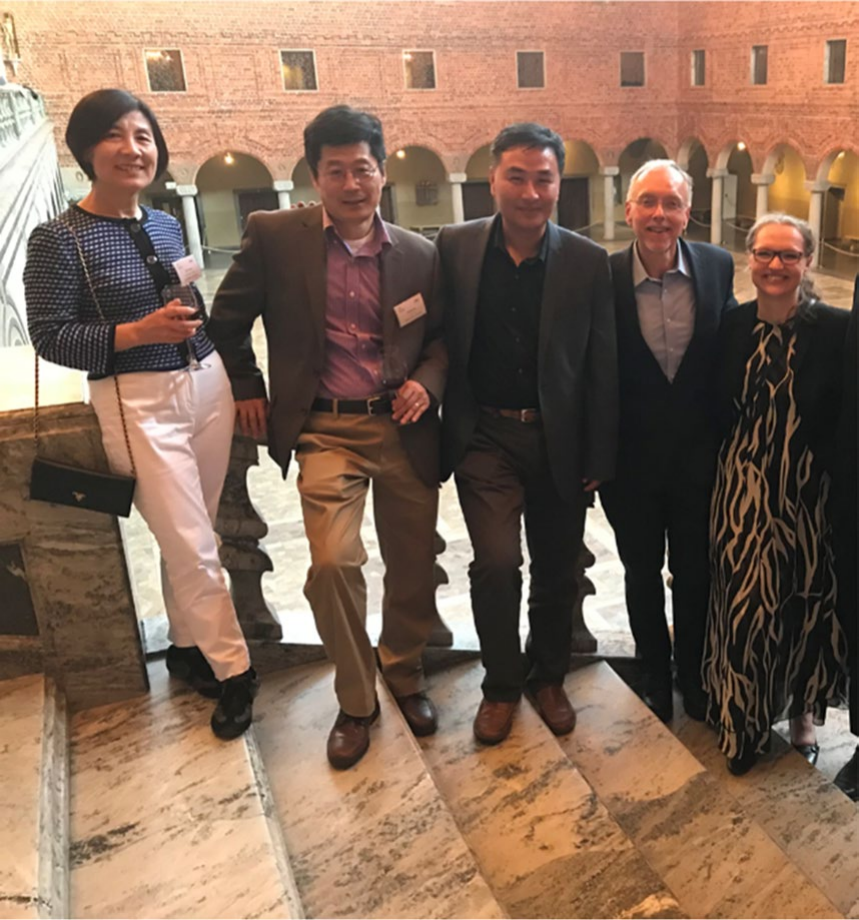



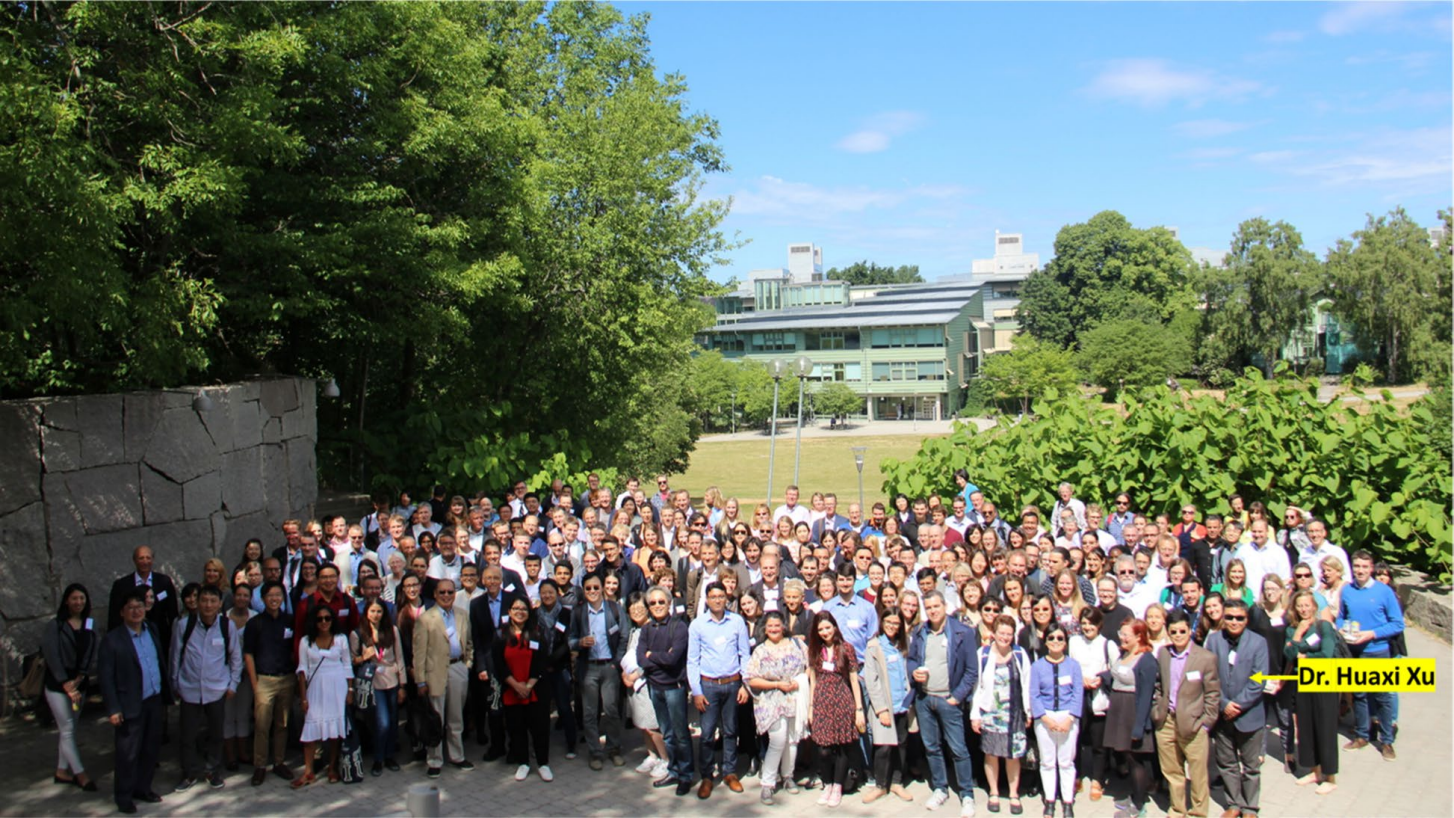


**Representative publications from Dr. Huaxi Xu**

1. **Xu, H.,** Gouras, GK., Greenfield, JP., Vincent, B, Naslund, J., Mazzarelli, L, Jovanovic, J. N, Seeger, M, Relkin, NR, Liao, F., Checler, F., Buxbaum, JD, Chait, BT., Thinakaran, G., Sisodia, S., Wang, R., Greengard, P., and Gandy, S. (1998). Estrogen reduces neuronal generation of Alzheimer beta-amyloid peptides. Nature Medicine 4(4):447–51. PMID: 9,546,791.

2. Dou, F., Netzer, WJ., Tanemura, K., Li, F., Hartl, FU., Takashima, A., Gouras GK., Greengard, P., and **Xu, H.** (2003) Chaperones increase association of tau protein with microtubules. Proc. Natl. Acad. Sci. USA 100(2):721–6. PMCID: PMC141063.

3. Zhang, YW, Wang, R., Liu, Q., Zhang, H., Liao, FF., and **Xu, H.** (2007) Presenilin/gamma-secretase-dependent processing of beta-amyloid precursor protein regulates EGF receptor expression. Proc. Natl. Acad. Sci. 104(25):10,613–8. PMCID: PMC1888796.

4. Zhang, YW, Wang, R., Liu, Q., Zhang, H., Liao, FF., and **Xu, H.** (2007) Presenilin/gamma-secretase-dependent processing of beta-amyloid precursor protein regulates EGF receptor expression. Proc. Natl. Acad. Sci. 104(25):10,613–8. PMCID: PMC1888796.

5. Zhang, YW., Liu, L., Zhang, X., Li, WB., Chen, Y., Huang, X., Sun, L., Luo, W., Netzer, WJ., Threadgill, R., Wiegand, G., Wang, R., Cohen, SN., Greengard, P., Liao, FF., Li, L., and **Xu, H.** (2009) A functional mouse retroposed gene Rps23r1 reduces Alzheimer’s beta-amyloid levels and tau phosphorylation. Neuron 64(3):328–40. PMCID: PMC3846276.

6. Wang, X., Zhao, Y., Zhang, X., Badie, H., Zhou, Y, Mu, Y, Loo, LS., Cai, L., Thompson, R., Yang, B., Chen, Y., Johnson, PF., Wu, C., Mobley, W., Zhang, D., Gage, FH., Ranscht, B., Zhang, YW., Lipton, SA., Hong, W., and **Xu, H.** (2013) Loss of sorting nexin 27 contributes to excitatory synaptic dysfunction by modulation of glutamate receptor recycling in Down’s Syndrome. Nature Medicine 19(4):473–80. PMCID: PMC3911880.

7. Tu, SC., Okamoto, S., Lipton, SA., **Xu, HX**. (2014) Oligomeric A beta-induced synaptic dysfunction in Alzheimer's disease. **Molecular Neurodegeneration **9:48. PMCID: PMC4237769

8. Zhao, Y., Tseng, IC., Heyser, CJ. Rockenstein, E., Mante M., Adame, A., Zheng, Q., Huang, T., Wang, X., Arslan, PE., Chakrabarty P., Wu, C., Bu, G., Mobley, WC., Zhang, YW., St. George-Hyslop, P., Masliah, E., Fraser, P., and **Xu, H.** (2015) Appoptosin-mediated caspase-cleavage of tau contributes to progressive supranuclear palsy pathogenesis. Neuron 87(5):963–75. PMCID: PMC4575284.

9. Zhu, B., Jiang L-L., Huang, T., Zhao, Y., Liu, T., Zhong, Y., Campos A., Pomeroy, K., Masliah, E., Zhang, D., and **Xu, H.** (2017) ER-associated degradation regulates γ-secretase activity, memory function and Alzheimer’s amyloid pathology. Nature Communications, 8(1):1472. PMCID: PMC5684335.

10. Huang, T., Zhao, Y., Jiang, L-L., Li, X., Liu, Y., Sun, Y., Pina-Crespo, J., Zhu, B., Masliah, E., Willnow, T.E., Pasquale, E.B., and **Xu, H.** (2017) SORLA attenuates EphA4 signaling and beta-amyloid-induced neurodegeneration. J. Exp. Med. 36:7996–8011. PMCID: PMC5716044.

11. Zhao, Y., Wu, X., Li, X., Jiang, L-L., Gui, X., Liu, Y., Sun, Y., Zhu, B., Pina-Crespo, C.C., Zhang, N., Chen, X., Bu, G., An, Z., Huang, T.Y., and **Xu, H.** (2018) TREM2 is a receptor for β-amyloid medicating microglial function. Neuron 97(5):1023–1031. PMCID: PMC5889092.

12. Jiang, L-L., Zhu, B., Zhao, Y., Li, X., Liu, T., Piña-Crespo, J.C., Zhou, L., Xu, W., Rodriguez, M.J., Yu, H.Y., Cleveland, D.W., Ravits, J.M., Da Cruz, S., Long, T., Huang, T.Y, and **Xu, H.** (2019) Membralin deficiency dysregulates astrocytic glutamate homeostasis leading to ALS-like impairment. J. Clin. Invest. 130:3103–3120. PMCID: PMC6668683.

13. Guo, TT., Zhang, DH., Zeng, YZ., Huang, TY.*, **Xu, HX**.*, Zhao, YJ.*, (2020) Molecular and cellular mechanisms underlying the pathogenesis of Alzheimer's disease. **Molecular Neurodegeneration** 15(1):40. *Co-corresponding author. PMCID: PMC7364557.

14. Liu, T., Zhu, B., Liu, Y., Zhang, X., Yin, J. Li, X., Jiang, L-L., Hodges, A., Rosenthal, S.B., Zhou, L., Yancey, J., Ms. McQuade, A., Blurton-Jones, M., Tanzi, R.E., Huang, T.Y.* and Xu, H.* (2020) Multi-omic comparison of Alzheimer's variants in human ESC-derived microglia reveals convergence at APOE. J. Exp. Med. 217 (12): e20200474, 2020. *Co-corresponding author. PMID: 32,941,599.

15. Zhu, B., Liu, Y., Hwang, S., Archuleta, K., Huang, HJ., Campos, A., Murad, R., Pina-Crespo, J., Xu, HX.*, Huang, TY.* (2022) Trem2 deletion enhances tau dispersion and pathology through microglia exosomes. **Molecular Neurodegeneration** 17(1):58. *Co-corresponding author. PMID: 32,941,599.

